# Use of multikinase inhibitors/lenvatinib in singular thyroid cancer scenarios

**DOI:** 10.1002/cam4.5154

**Published:** 2022-10-06

**Authors:** Carles Zafón, Beatriz Castelo

**Affiliations:** ^1^ Department of Endocrinology Vall d'Hebron Hospital Campus Barcelona Spain; ^2^ Department of Medical Oncology University Hospital La Paz Madrid Spain

## Abstract

Thyroid cancer is the most frequent endocrine tumor. In locally advanced or metastatic disease there are only two types of treatment available: radioactive iodine (RAI) while the disease is RAI‐sensitive and multikinase inhibitors, lenvatinib and sorafenib, when the disease becomes RAI‐refractory. The objective of this publication is to review the current knowledge on the use of targeted therapy and the specific practical considerations concerning lenvatinib in the treatment of patients with differentiated thyroid cancer under special circumstances.

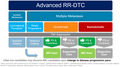

Thyroid cancer is the most frequent endocrine tumor.^1^ In locally advanced or metastatic disease there are only two types of treatment available: radioactive iodine (RAI) while the disease is RAI‐sensitive and multikinase inhibitors (MKIs), lenvatinib and sorafenib, when the disease becomes RAI‐refractory.

In RAI refractory disease, daily clinical practice entails multiple treatment situations that, in many cases, are not represented in the controlled registration trials, post‐authorization phase IV trials, or retrospective studies. This situation is even more frequent when the pathology is rare, such as for radioiodine‐refractory differentiated thyroid cancer (RR‐DTC). Although none of the currently approved drugs have been systematically evaluated in unusual clinical scenarios, some anecdotal experience and case reports have been published.

Provided lenvatinib is the latest medication approved for the management of RR‐DTC, there is scant, or no, data on its use in less frequent circumstances involving patients with relevant comorbidities or with disease localization requiring concomitant therapeutic approaches. Altogether, these scenarios represent challenging clinical issues where the available evidence should be combined with expertise in the management of RR‐DTC patients, always keeping in mind what are the therapeutic goals to pursue in each case. The objective of this publication is to review the current knowledge on the use of targeted therapy and the specific practical considerations concerning lenvatinib in the treatment of patients with DTC under special circumstances.

The publication is divided into 8 chapters. Four chapters review the use of multikinase inhibitors (MKIs)/lenvatinib in patients with important comorbidities and the other four review the use of MKIs/lenvatinib in concomitance with other therapies that are sometimes necessary for the treatment of metastatic or locoregional RR‐DTC.

Renal and hepatic impairments in patients with cancer are major issues for drug dosage drug‐related adverse events. This scenario is reviewed in the first article. According to the available scientific evidence, Martinez‐Trufero summarizes a series of recommendations for initiating MKIs in patients affected by these conditions. Some of the most common toxicities described with sorafenib and lenvatinib are directly linked to renal and/or hepatic function. Hence, both organs must be carefully monitored, not only in patients with previously compromised status, but also in patients without any diagnosed function impairment. Although there is scarce literature regarding the management of these drugs in thyroid cancer, Martinez‐Trufero proposes some useful tips for patients with kidney or liver disease who must be treated with MKIs. Finally, a short clinical case illustrates how to treat a patient with iodine‐refractory follicular thyroid cancer affected by both renal and hepatic impairment during lenvatinib treatment.

Another major topic is summarized in the second article of this section: MKI treatments in immunosuppressed patients. Treatment improvement in several immunosuppression‐associated diseases such as solid organ transplantation or HIV/AIDS has made possible longer survival of these patients, and so the possibility to develop de novo malignancies. There is very little data on immunodeficient patients with advanced thyroid cancer treated with MKIs. Therefore, Basté Rottlan focused on analyzing the scientific evidence in cases of post‐liver transplantation recurrence of hepatocellular carcinoma, a situation described in around 20% of patients. MKIs such as sorafenib and lenvatinib have been used in this clinical scenario, providing some interesting conclusions. For instance, it seems that immunosuppressed patients are more susceptible to MKI‐related side effects, while it is still the only current alternative provided immunotherapy is contraindicated in patients at high risk of transplant rejection or life‐threatening flaresfrom an underlying autoimmune disease. Accordingly, Basté Rottlan includes a series of treatment recommendations that could be applied to immunosuppressed patients with advanced thyroid cancer.

A more common clinical condition is addressed in the article from Jimenez‐Fonseca. Cardiovascular disease is the leading cause of morbidity and mortality worldwide. Moreover, cardiovascular risk factors (arterial hypertension, obesity, diabetes, smoking) are present in many adults, becoming the most frequent cancer‐associated comorbidity. As vascular endothelial growth factor inhibitors sorafenib and lenvatinib induce vasoconstriction, being the cause of arterial hypertension, this is one of the most common side effects of both drugs. In accordance, cardiovascular vulnerability is a major feature in oncological patients. With the aim of minimizing that risk, Jimenez‐Fonseca provides detailed recommendations on when thyroid cancer patients must be treated with MKIs, with special focus on control of hypertension, proteinuria, and electrolyte disorders.

The last scenario in this first section addresses an increasingly frequent situation, that is the management of MKI treatment in patients with other primary malignant neoplasms. As Sambo explains in his review, it is expected that patients with multiple primary malignancies will increase in the future. This scenario is not rare in thyroid cancer patients. MKIs approved for RR‐DTC are also indicated for the treatment of many other tumors. However, scientific data on their potential usefulness to treat several tumors with the same drug are scarce. Sambo reviews the evidence published so far, with special emphasis on treatment with MKIs in specific combinations of malignancy and the risk of developing second primary tumors as an adverse effect of MKI treatment.

Regarding the use of MKIs/lenvatinib in patients with RR‐DTC that has spread to the central nervous system, Alonso‐Gordoa advises that promptly initiating and maintaining MKI therapy along the treatment pathway is vital for disease control, in the same necessary to correctly stop and restart treatment when specific procedures are to be performed.

There is very limited data on the management of MKIs/lenvatinib concomitant with locoregional therapies for the treatment of RR‐DTC patients. However, Berciano‐Guerrero has provided a set of practical directions on the use of surgery, embolization, enolization, radiofrequency ablation, or radiotherapy for the management of localized disease, while also accounting for the wide heterogeneity of clinical presentations and overlapping indications among the procedures reviewed.

Probably the use of MKIs/lenvatinib concomitant with radioiodine for the treatment of RR‐DTC is a frequent topic for debate in daily practice but, unfortunately, there is still little evidence in the literature that demonstrates the possibility of re‐sensitization to RAI in locoregional or metastatic disease. Despite this, Anido‐Herranz provides an updated dissertation of the state‐of‐the‐art in this situation and the potential clinical scenarios where either sequential or simultaneous use of these therapies could be feasible amidst the various controverted aspects.

Bone is the second most frequent metastatic localization.^2^ So, to review the use of MKIs/lenvatinib concomitant with antiresorptive therapy for the treatment of RR‐DTC is of utmost importance. In this chapter, Navarro‐Gonzalez walks us through the prognostic impact that this therapy has on bone‐related disorders in the context of cancer metastases, including prevention of skeletal‐related events (e.g. bone pain and fractures) and key caveats to consider previous to starting and while continuing treatment with these therapies, such as the importance of oral care to reduce the risk of osteonecrosis of the jaw, potentially increased by the antiangiogenic effect of MKIs such as lenvatinib.

We hope this dedicated supplement with expert recommendations on the management of MKIs/lenvatinib across various singular clinical scenarios coexisting with the radioiodine‐refractory disease, will help colleagues better orient their daily decisions and encourage best‐practice sharing among the thyroid cancer community.

## FUNDING INFORMATION

The authors received honoraria payment from Eisai Farmacéutica SA in line with ICMJE guidelines.

## CONFLICT OF INTEREST

Dr Carles Zafón has received research funding, honoraria, and non‐financial or other support from Sanofi and Eisai. Dr Beatriz Castelo has received research funding, honoraria, and non‐financial or other support from Sanofi, EISAI, BMS and Merck.
